# Wide Variation in Absolute Cardiovascular Risk Assessment in Aboriginal and Torres Strait Islander People with Type 2 Diabetes

**DOI:** 10.3389/fpubh.2016.00037

**Published:** 2016-03-08

**Authors:** Bhakti R. Vasant, Veronica Matthews, Christopher P. Burgess, Christine M. Connors, Ross S. Bailie

**Affiliations:** ^1^Menzies School of Health Research (Brisbane Office), Brisbane, QLD, Australia; ^2^Top End Health Services, Primary Health Care Branch, Northern Territory Government, Casuarina, NT, Australia

**Keywords:** absolute cardiovascular risk assessment, indigenous, diabetes, audit, quality improvement, primary health care

## Abstract

**Background:**

Absolute cardiovascular risk assessment (CVRA) is based on the combined effects of multiple risk factors and can identify asymptomatic individuals at high risk of cardiovascular disease (CVD). Aboriginal and Torres Strait Islander people, the Indigenous people of Australia, are disproportionately affected by CVD and diabetes. Our study aimed to investigate variations in the use of absolute CVRA in patients with diabetes at Indigenous community healthcare centers and to identify patient and health center characteristics that may contribute to this variation.

**Methods:**

Audits of clinical records of 1,728 patients with a known diagnosis of diabetes across 121 health centers in four Australian States/Territories [Northern Territory (NT), South Australia, Western Australia, and Queensland] over the period 2012–2014 were conducted as part of a large-scale continuous quality improvement program. Multilevel regression modeling was used to quantify variation in recording of CVRA attributable to health center and patient characteristics.

**Results:**

The proportion of eligible patients with documented CVRA was 33% (*n* = 574/1,728). The majority (95%) of assessments were conducted in the NT. Multilevel regression analysis showed health center characteristics accounted for 70% of the variation in assessments in the NT. Government-operated health centers had 18.8 times the odds (95% CI 7.7–46.2) of recording CVRA delivery compared with other health centers.

**Conclusion:**

Health centers in the NT delivered the majority of absolute CVRA to Indigenous patients with diabetes in our study. Health systems factors that may have facilitated provision of CVRA in the NT include decision support tools and a reporting process for CVRA delivery. Implementation of similar systems in other jurisdictions may help improve CVRA delivery. Early identification and treatment of high risk individuals through wider use of CVRA may help reduce the burden of CVD in Indigenous Australians with diabetes.

## Introduction

Absolute cardiovascular risk assessment (CVRA) provides an assessment of cardiovascular risk based on the effects of a combination of risk factors. CVRA helps identify individuals who are asymptomatic but at high risk of cardiovascular disease (CVD), including stroke, transient ischemic attack, myocardial infarction, peripheral arterial disease, and heart failure (1). Early identification of individuals at high risk of CVD (>15% risk of CVD within the next 5 years) using CVRA can enable effective management of modifiable risk factors, including lifestyle changes, pharmacotherapy, and improvement of CVD-related clinical targets (1–8). CVRA and targeted treatments are cost-effective and have the potential to reduce the burden of CVD (9, 10).

Globally, CVD is the leading cause of death and imposes a substantial social and economic burden at population and household levels (9, 11). Socioeconomically disadvantaged populations, including Indigenous peoples, have a disproportionate burden of CVD and risk factors of CVD, including diabetes (12–14). In Aboriginal and Torres Strait Islander people, the Indigenous people of Australia, CVD and diabetes, respectively, account for 27 and 8% of deaths (15). CVD and Type 2 diabetes also account for 23 and 12%, respectively, of health gaps between Indigenous and non-Indigenous Australians (16). Preventive efforts targeting modifiable risk factors for both CVD and diabetes offer significant opportunity to improve Indigenous health in Australia and internationally (12–14, 16). A recent National Health and Medical Research Council (NHMRC) document on improving performance in the management of CVD in Australian hospitals noted that significant improvements in cardiovascular health care delivery are possible, including provision of preventive services such a CVRA to Aboriginal and Torres Strait Islander people (17).

The National Vascular Disease Prevention Alliance (NVDPA) recommends the Framingham Risk Equation (derived from the Framingham Heart Study) be used to calculate absolute CVD risk and includes age, sex, systolic blood pressure, smoking status, total cholesterol, HDL cholesterol, diabetes, and electrocardiogram evidence of left ventricular hypertrophy in CVRA calculations (1). NVPDA recommends that “adults with diabetes who are 60 years or less” without known CVD or increased risk of CVD should receive absolute CVRA at least every 2 years depending on the level of risk of CVD (1). Although the Framingham Risk Equation has been found to underestimate risk in Aboriginal and Torres Strait Islander people and in people with diabetes, absolute CVRA using the Framingham risk equation is a more accurate predictor of future vascular events for these populations than any single CVD risk factor alone (1, 18, 19).

The use of absolute CVRA in the primary care setting for Aboriginal and Torres Strait Islander people with diabetes has not been previously reported. Previous studies have been limited to assessment of individual risk factors and the measures taken to address these risk factors (20–23). The aim of our study is to investigate the delivery of absolute CVRA in patients with Type 2 diabetes attending Indigenous primary healthcare services between 2012 and 2014 and to identify the influence of health center and individual patient level factors on the delivery of absolute CVRA.

## Materials and Methods

From 2002, the Audit and Best Practice for Chronic Disease (ABCD) project has been operating across several jurisdictions and aims to investigate variation in and to improve the quality of care in a range of priority areas in Indigenous primary care, including chronic diseases, maternal health, and child health. From 2010, One21seventy, a not-for-profit entity, continued the service support for continuous quality improvement (CQI) to over 200 Indigenous health centers (24). These health centers use clinical audit tools developed by the Menzies School of Health Research to assess recording of service delivery and quality of service provision. One hundred and seventy of these services have voluntarily provided their de-identified audit data to the ABCD National Research Partnership for research of variation in quality of care, barriers, and strategies for improvement.

This study used the ABCD/One21seventy Type 2 diabetes audit dataset. This dataset included records of participating health centers’ delivery of recommended services for patients with Type 2 diabetes. Of 170 Aboriginal and Torres Strait Islander community health centers participating in the ABCD National Research Partnership, 121 health centers in four states and territories audited clinical records to assess the quality of Type 2 diabetes care between January 2012 and December 2014. For the participating community health centers, the records of Indigenous patients who met the following criteria were eligible for audit: (1) A definite diagnosis of Type 2 diabetes according to health center records; (2) aged 15 years and above; and (3) lived in the community for 6 months or more in the last year. Residence in the community for ≥6 months in the last year was determined by examining health center records and discussions with Aboriginal and/or Torres Strait Islander Health Workers/Practitioners familiar with the community.

From December 2011, the diabetes audit tool included a question on CVRA delivery, “Is there a record that an absolute CVR assessment has been performed within the last 12 months?” (25). The audit protocol specified that there needed to be a clear record that a CVRA had been completed. While a record of a statement such as “CVRA done” was sufficient to satisfy this requirement, there was also usually a record of level of risk. To determine current status of absolute CVRA delivery, records from the most recent Type 2 diabetes audit between 2012 and 2014 from each health center were included. Data were abstracted by health center staff using standard protocols and who had been trained in the use of ABCD/One21seventy audit tools, with support from quality improvement facilitators and One21seventy staff.

We excluded records of patients greater than 60 years of age in line with the NVDPA recommendations (1, 26). Patients with chronic heart disease, congestive heart failure, systolic blood pressure greater than 180 mmHg, or diastolic blood pressure greater than 110 mm Hg and previous acute myocardial infarction were excluded from the study because they have clinically determined high risk of CVD (1). Patients with estimated glomerular filtration rate (eGFR) less than 45 mL/min/1.73 m^2^ have clinically determined high risk of CVD (1). Categories of eGFR included in the audit tool were ≥60 mL/min/1.73 m^2^, 30–59 mL/min/1.73 m^2^, 15–29 mL/min/1.73 m^2^, <15 mL/min/1.73 m^2^, and “no record.” As <45 mL/min/1.73 m^2^ was not specifically included as a category of eGFR in the audit tool, we excluded patients with eGFR <60 mL/min/1.73 m^2^ because of likely high CVD risk.

Health center characteristics included in the audit tool were size of service population, type of health center (community controlled or government operated), CQI experience, and location based on the Australian Standard Geographical Classification System (very remote, remote or non-remote). The classification of remoteness is based on the physical distance of a populated locality to the nearest urban center and reflects access to goods and services (27). Patients were excluded from the analysis if they did not attend the health center over the past 12 months or if CVRA was recorded as “not due.”

The audit records included the following patient-level characteristics: age, sex, documented comorbid conditions, and complications. Hypertension, dyslipidemia, chronic obstructive pulmonary disease (COPD), chronic kidney disease (CKD), depression, and other mental illness were each recorded as present or absent. Similarly, complications of diabetes (retinopathy, neuropathy, foot ulcer, amputation, and gastroparesis) were recorded as present or absent.

Ethics approval was obtained from research ethics committees in each jurisdiction: Human Research Ethics Committee of the Northern Territory Department of Health and Menzies School of Health Research (HREC-EC00153); Central Australian Human Research Ethics Committee (HREC-12-53); Queensland Human Research Ethics Committee Darling Downs Health Services District (HREC/11/QTDD/47); South Australian Aboriginal Health Research Ethics Committee (04-10-319); Curtin University Human Research Ethics Committee (HR140/2008); Western Australian Country Health Services Research Ethics Committee (2011/27); Western Australia Aboriginal Health Information and Ethics Committee (111-8/05); and University of Western Australia Human Research Ethics Committee (RA/4/1/5051).

### Statistical Analysis

Our data had a multilevel dependency structure, with individual patients clustered within health centers and health centers clustered within jurisdictions. Multilevel mixed effects logistic regression analysis was used to quantify variation in the delivery of absolute CVRA attributable to health center and patient level characteristics. We calculated unadjusted odds ratios to measure the unadjusted association between dependent and independent variables. All variables, including non-significant variables, were included in further analyses. In a hierarchical approach, we included health center variables (Model A) and then patient variables (Model B). The amount of variation due to introduction of the different groups of variables in the models was determined by the proportional change in variance at different levels. Because CVRA delivery outside the Northern Territory (NT) was limited, we restricted our analysis to this jurisdiction and compared results to the same analysis for the NT and other states. A *p* value ≤0.05 was considered significant. We completed statistical analyses with STATA software, version 14.

#### Sensitivity Analyses

We evaluated the inclusion of patients with CKD *and* eGFR greater than 60 mL/min/1.73 m^2^, that is separate analyses were conducted for datasets including (1) patients with CKD *and* eGFR greater than 60 mL/min/1.73 m^2^ and (2) patients with no evidence of CKD or eGFR greater than 60 mL/min/1.73 m^2^.

## Results

**Figure 1:**
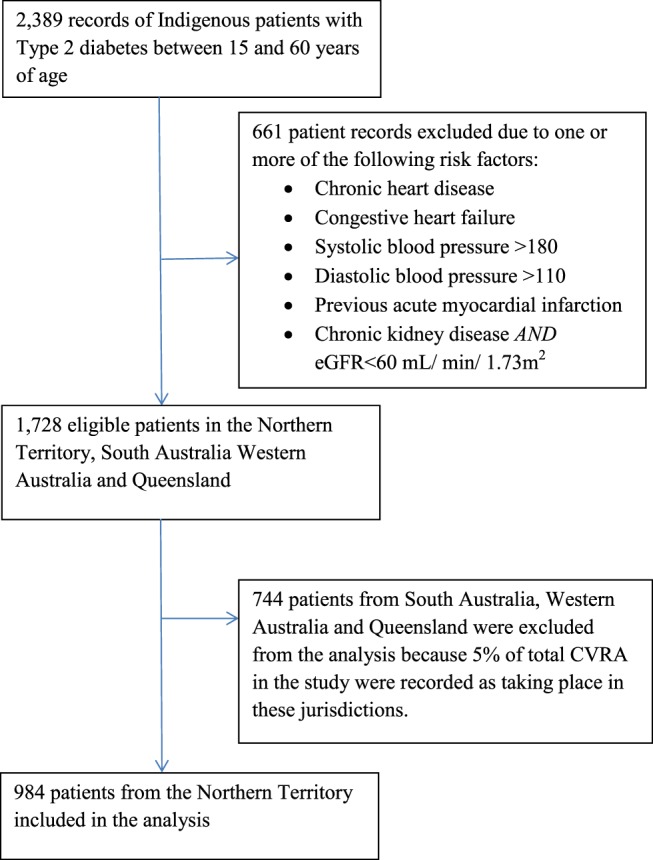
**Enrollment of Aboriginal and Torres Strait Islander patients with Type 2 diabetes into the study**.

The participating health centers had 2,389 clinical records for Indigenous patients with Type 2 diabetes between 15 and 60 years of age. The records for 1,728 patients met the inclusion criteria for the study (Figure 1). There was wide variation in delivery of absolute CVRA across jurisdictions (Table 1). In the NT, 56% of eligible patients received CVRA, compared to 3% in other jurisdictions. The majority (95%) of assessments were conducted in the NT.

**Table 1:** **Number and percentage of patients who received absolute CVRA in participating health centers between 1 January 2012 and 31 December 2014 in the Northern Territory and other jurisdictions**.

State	Number of patients who received absolute CVRA (percentage of eligible patients in state)	Total number of eligible patients	Number of health centers
Northern Territory	548 (56)	984	52
Other jurisdictions^a^	26 (3)	744	69
Total	574 (33)	1,728	121

*^a^Other jurisdictions include Queensland, South Australia, and Western Australia*.

Of the 52 participating health centers in the NT, 87% were located in very remote areas, 52% had a service population of 500 or fewer, and 77% were government operated (Table 2). The proportion of government and community-controlled health centers were estimated to be 76 and 52%, respectively, of total government and community-controlled health centers in the NT. Eight hundred and seventy seven patients (89%) from the NT were between 30 and 60 years of age (Table 2). Sixty-two percent of patients were females. Fifty-one percent of patients were recorded as having dyslipidemia. Diabetic neuropathy was the most common complication of diabetes and was recorded for 4% of patients.

**Table 2:** **Health center and patient characteristics of participating health centers in the Northern Territory and other jurisdictions between 1 January 2012 and 31 December 2014**.

	Characteristic	Northern Territory		Other jurisdictions	
		Total number of health centers/patients	Number of patients recorded as receiving CVRA (percentage in brackets)	Total number of health centers/patients	Number of patients recorded as receiving CVRA (percentage in brackets)
**Health center (*N* = 121)**					
Location	Non-remote	2	9 (32)	15	10 (7)
	Remote	5	102 (61)	5	2 (6)
	Very remote	45	437 (55)	49	14 (2)
Type of health center	Community-controlled	12	64 (24)	8	2 (2)
	Government	40	484 (68)	61	24 (4)
Service population	≤500	27	263 (64)	28	13 (5)
	501–999	10	119 (56)	16	2 (1)
	≥1000	15	166 (47)	25	11 (4)
CQI experience	Nil previous cycles	3	44 (50)	10	5 (7)
	1–2 previous cycles	23	285 (66)	33	6 (2)
	≥3 previous cycles	26	219 (47)	26	15 (4)
**Patient (*N* = 1,728)**					
Age group	15 to <30	107	50 (47)	43	2 (5)
	30 to <45	434	240 (55)	244	6 (2)
	45–60	443	258 (58)	457	18 (4)
Sex	Male	375	200 (53)	313	9 (3)
	Female	609	348 (57)	431	17 (4)
Comorbidities	Hypertension	338	204 (60)	312	15 (5)
	COPD	126	66 (52)	89	4 (4)
	Dyslipidemia	502	285 (57)	365	12 (3)
	CKD	327	211 (65)	59	1 (2)
	Depression	51	25 (49)	56	3 (5)
	Other mental illness	43	22 (51)	39	2 (5)
Complications	Retinopathy	36	22 (61)	64	2 (3)
	Neuropathy	40	16 (40)	36	1 (3)
	Foot ulcer	14	9 (64)	23	1 (4)
	Amputation	11	7 (64)	5	0 (0)
	Gastroparesis	6	0 (0)	13	0 (0)

The strongest predictor of CVRA delivery was location of health center in the NT. (Table A1 in Supplementary material). For the NT, unadjusted logistic regression showed that government-operated health centers were more likely to record delivery of CVRA than other health centers (unadjusted odds ratio 13.6, 95% confidence interval 6.1–30.5). In the adjusted analysis (Model A), the association between government-operated health centers and CVRA delivery increased (OR 21.0, 95% CI 8.7–50.4). Remote location was also associated with provision of CVRA (OR 12.3, 95% CI 1.6–93.5) in Model A. The proportional change in variance from the unadjusted model to Model A showed that health center factors explained 70% of variation in the administration of absolute CVRA across health centers in the NT (Table 3). The addition of patient level variables did not appreciably explain further variation in CVRA. Remote location (OR 13.0, 95% CI 1.6–103.7), government-operated health centers (OR 18.8, 95% CI 7.7–46.3), and female gender (OR 1.5, 95% CI 1.1–2.1) were significantly associated with CVRA delivery in Model B.

**Table 3:** **Unadjusted and adjusted multilevel regression analysis of health center and patient level characteristics on recording of absolute CVRA for patients with diabetes in participating health centers in the Northern Territory between 1 January 2012 and 31 December 2014 (*n* = 52 health centers; 984 patient records)**.

Predictors		Unadjusted odds ratio			Empty model	Model A – health center characteristics only			Model B – health center and patient characteristics		
					
		UOR	95% CI	*p* value		OR	95% CI	*p* value	OR	95% CI	*p* value
**Health center characteristics**											
Location	Non-remote	1	(Base)			1	(Base)		1	(Base)	
	Remote	2.96	0.20–43.52	0.428		12.38	1.64–93.48	0.015^a^	12.98	1.63–103.66	0.016^a^
	Very remote	2.43	0.23–25.76	0.460		1.77	0.31–10.28	0.524	1.61	0.26–9.84	0.604
Type of health center:	Community controlled	1	(base)	0.000^a^		1	(base)	0.000^a^	1	(base)	0.000^a^
	Government	13.63	6.09–30.54			19.67	8.21–47.16		18.84	7.67–46.29	
Service population	≤500	1	(base)			1	(base)		1	(base)	
	501–999	0.62	0.20–1.90	0.402		2.03	0.88–4.73	0.099	2.07	0.88–4.90	0.228
	≥1000	0.38	0.14–1.02	0.555		0.66	0.32–1.36	0.258	0.65	0.31–1.38	0.262
CQI experience	Nil previous cycles	1	(base)			1	(base)		1	(base)	
	1–2 previous cycles	2.57	0.40–16.38	0.318		1.11	0.31–3.95	0.876	1.07	0.29–3.88	0.924
	≥3 previous cycles	1.01	0.16–6.33	0.991		1.10	0.32–3.82	0.877	1.06	0.30–3.76	0.928
**Patient characteristics**											
Sex	Male	1	(base)						1	(base)	0.019^a^
	Female	1.40	1.01–1.93	0.038^a^					1.49	1.07–2.07	
Age group	15 to <30	1	(base)						1	(base)	
	30 to <45	1.19	0.71–2.00	0.512					1.14	0.67–1.94	0.629
	45–60	1.40	0.83–2.35	0.210					1.34	0.77–2.32	0.306
Comorbidities^b^	Hypertension	1.08	0.77–1.51	0.659					0.94	0.65–1.36	0.738
	COPD	0.90	0.56–1.44	0.663					0.85	0.52–1.36	0.490
	Dyslipidemia	1.22	0.89–1.68	0.218					1.25	0.89–1.76	0.197
	CKD	1.41	0.99–2.00	0.059					1.29	0.89–1.86	0.178
	Depression	0.82	0.40–1.65	0.573					0.87	0.41–1.84	0.723
	Other mental illness	0.64	0.30–1.35	0.241					0.66	0.30–1.45	0.305
Complications^b^	Retinopathy	1.06	0.47–2.42	0.886					0.98	0.42–2.27	0.960
	Neuropathy	0.69	0.32–1.48	0.336					0.70	0.32–1.55	0.379
	Foot ulcer	2.07	0.52–8.22	0.301					2.26	0.54–9.43	0.261
	Amputation	1.51	0.28–8.00	0.629					1.44	0.27–7.64	0.666
	Gastroparesis^c^	1	(Empty)						1	(Empty)	
Random effects (intercepts)	Variance (SE)				2.22 (0.58)	0.66	(0.22)		0.69	(0.23)	
	Proportional change in variance					70.09%			68.86%		

*^a^Statistically significant*.

*^b^Comorbidities and complications were compared with patients without the specific comorbidity or complication, such that an odds ratio of 1 relates to not having the specific comorbidity or complication*.

*^c^The six patients with gastroparesis had no recorded CVRA*.

### Sensitivity Analyses

When patients with CKD *and* eGFR greater than 60 mL/min/1.73 m^2^ were excluded, predictors of CVRA delivery were similar to the results described above. (Table A2 in Supplementary material)

## Discussion

Our study found wide variation in the delivery of absolute CVRA across jurisdictions over the study period. The majority (95%) of assessments were conducted in the NT. Health center factors accounted for 70% of the variation in CVRA delivery in the NT. Factors associated with increased recording of CVRA delivery were government-operated health centers, remote health centers, and female gender (Model B, Table 3).

Men represented 38% of eligible patients in the NT and were less likely to receive CVRA than women. While fewer Aboriginal and Torres Strait Islander men than women report a diagnosis of diabetes (7.3% compared to 8.9%) (28), they also access preventive health services less often than women (29, 30). Poor health seeking among men results in poor use of health services and limited opportunities for access to health information, promotion, and preventive care. Further research is required to better understand how to deliver preventive health services and gender-appropriate healthcare to men (29).

Consistent with other studies, we found gaps in recording absolute cardiovascular risk (6, 19, 31). The Treatment of Cardiovascular Risk using Electronic Decision Support (TORPEDO) study recorded relatively high levels of CVRA. In this study, 30 primary healthcare centers in Queensland and New South Wales were randomized to receive a computer-guided quality improvement intervention, and 30 centers were randomized to usual care. At intervention sites, 63% of eligible patients received appropriate CVD screening compared to 53% of patients attending non-intervention sites (RR 1.25; 95% CI 1.04–1.50; *p* = 0.02) (32). In the Kanyini Audit, 53% of 1,165 randomly selected case records of adults attending Indigenous health centers lacked information for one or more Framingham risk variables. Screening and management gaps were similar to those found in non-Indigenous health settings, suggesting a need for improvement across the health system (19). Despite similarities in the gaps in screening, ongoing disparities in cardiovascular risk and complications persist between Indigenous and non-Indigenous Australians (16, 22). Absolute CVRA may help reduce these disparities by encouraging early detection and intervention for Indigenous adults at high risk of CVD (6, 22).

There are a number of possible explanations for relatively high delivery of CVRA in government-operated health centers in the NT. First, Burgess et al.’s study of CVRA in Arnhem Land in 2005 showed that CVRA during the adult health check may contribute to better and earlier detection of cardiovascular risk and population reductions in cardiovascular risk (6). These findings led to the incorporation of CVRA into the Central Australian Rural Practitioner’s Association (CARPA) Manual (33). Second, following the study in Arnhem Land, CVRA was promoted in Preventable Chronic Disease Strategy Workshops in the NT in 2006–2007. Primary care providers participating in the workshop received education about the utility of CVRA in the adult health check (34). Chronic care educators and CQI facilitators have continued to provide outreach support to primary care providers to implement CVRA. Third, following the commencement of the Chronic Conditions Management Model (CCMM) in 2012, Northern Territory Government (NTG) health services received regular feedback about (i) the proportion of Indigenous patients aged >20 years who received CVRA, (ii) gaps between screening and initiating treatment to allow patients who are missing care to be identified and recalled, and (iii) achieving targets for modifiable risk factors for individuals identified at high risk of CVD. Burgess et al. found that implementation of CCMM led to improvements in NT population coverage of CVRA from 23% in mid-June 2012 to 58.5% in August 2014 (8). Introducing similar auditing processes and national CVRA-related key performance indicators may improve CVRA delivery in other jurisdictions.

Wide-scale CVRA can be supported by including a user-friendly CVRA calculator in clinical information systems. For example, the computer-guided decision support tool in the TORPEDO study improved CVD risk measurement. The absence of an electronic CVRA calculator has been identified as a barrier to CVRA delivery in primary care settings (35). Community-controlled health centers in the NT have campaigned for incorporation of a CVRA calculator in clinical information systems but this is not yet available. As a result, clinicians calculate CVRA manually. Lack of access to such a tool may contribute to underreporting of CVRA in community-controlled health centers in the NT.

There is contention about the usefulness of CVRA in Aboriginal and Torres Strait Islander people and people with diabetes because the Framingham risk equation can underestimate risk in these populations (1, 18). To date, no studies have been published evaluating adjustments for Indigenous Australians that are similar to adjustments for Maori, Pacific Islander, and Indian patients recommended in New Zealand guidelines (6, 36). While further work is required to develop accurate CVD risk estimates for Indigenous Australians, adjustments included in the CARPA Manual risk calculators may provide a more reliable estimate of CVD risk than the Framingham risk equation (6, 33). Further research is also required to assess whether CVRA leads to improved clinical outcomes in Indigenous people with diabetes.

The strength of this study is the inclusion of 76% of NT government-operated services and 52% of community-controlled health services in this study. As a significant proportion of NT health services are included in this audit, the results are likely to be generalizable to the NT, and perhaps to a more limited extent to other jurisdictions. The age groups of patients included in the study are similar to the age groups for which Aboriginal and Torres Strait Islander adult health checks are recommended (aged 15 years and over but under the age of 55 years) (37). Our study assessed delivery of CVRA in regular residents in the community (i.e., individuals who had lived in the community for at least six of the past 12 months) as visitors or short-term residents of the community may not be regarded by health center staff as being within the priority target group for preventive care.

The main limitations of this study are (1) potential participation bias as only community health centers that participated in the One21seventy clinical audit cycles were included in the study. For example, the TORPEDO study found relatively high levels of CVRA in community controlled health services in Queensland (32). Less than 5% of community controlled health services in Queensland participated in this study. (2) The study relied on patient records to abstract information about CVRA. Under-documentation may result in underestimation of service delivery and contribute to over-servicing, inefficiency and poor coordination and continuity of patient care. However, clear, accurate documentation is essential for coordinating health service delivery (23). (3) Clinical audits were carried out by multiple data abstractors and inter-rater reliability was not formally assessed in this study. Although inter-rater reliability was satisfactory in previous studies that used similar audit forms (23, 38), it is possible that manual CVRA calculations may have been missed. (4) The ABCD audit protocol did not record total cholesterol >7.5 mmol/L, familial hypercholesterolemia, microalbuminuria, or eGFR <45 mL/min/1.73 m^2^. Adjustments to the audit protocol may improve detection of these patients with clinically determined high risk of CVD in future studies.

While the clinical audit included information on the date of diagnosis of diabetes, in many cases there was not a clear record of date of diagnosis. We therefore included all patients with a diagnosis of diabetes, regardless of the date of diagnosis. There may therefore be a small proportion of patients who may have been diagnosed so recently that the period between the diagnosis and the date of the audit may have been so short that it would not be reasonable to expect that some of the scheduled services specified in the audit tool would have been delivered. The same approach was applied in all audits, and so comparisons between groups are unlikely to be affected. The proportion of patients affected is also likely to be too small to have made a meaningful impact on the findings. Despite limitations of the audit method, the study provides valuable guidance for policy, practice and further research.

## Conclusion

In conclusion, we found wide variation in the delivery of CVRA across jurisdictions in our study. The NT delivered the majority of CVRA to Aboriginal and Torres Strait Islander patients with diabetes. Health center characteristics accounted for the majority of variation in CVRA delivery. A number of health systems factors, including the electronic CVRA calculator and the CCMM, may have facilitated delivery of this service. Further understanding of the impact of CVRA on the clinical outcomes of Aboriginal and Torres Strait Islander people with diabetes may provide additional guidance to clinicians and policy makers. Efforts to improve early identification and treatment of high risk individuals in the primary care setting may play a critical role in reducing the burden of CVD in Indigenous Australians with diabetes.

## Author Contributions

BV completed a literature review for this article, drafted the project proposal, performed the statistical analysis, and drafted the manuscript. VM manages the ABCD dataset and provided assistance with the statistical analysis and drafting of the manuscript. RB is responsible for overall leadership of the ABCD National Research Partnership, helped develop the project proposal, provided feedback for the literature review, and assisted with drafting the manuscript. CB and CC assisted with interpretation of the analysis and drafting the manuscript.

## Conflict of Interest Statement

The authors declare that the research was conducted in the absence of any commercial or financial relationships that could be construed as a potential conflict of interest.

## Abbreviations

ABCD, audit and best practice for chronic disease; CARPA, Central Australian Rural Practitioner’s Association; CCMM, chronic conditions management model; CKD, chronic kidney disease; CQI, continuous quality improvement; CVD, cardiovascular disease; CVRA, cardiovascular risk assessment; NHMRC, National Health and Medical Research Council; NT, Northern Territory; NTG, Northern Territory government; NVPDA, National Vascular Disease Prevention Alliance; PCIS, primary care information system.
