# Supplementary material for: Wide Variation in Absolute Cardiovascular Risk Assessment in Aboriginal and Torres Strait Islander People with Type 2 Diabetes
Source: Front Public Health. 2016 Mar 8;4:37. doi: 10.3389/fpubh.2016.00037 (PMC4781864; doi:10.3389/fpubh.2016.00037)
Supplement: Supplementary file 1 [file Data_Sheet_1.PDF]

Appendix 1: Unadjusted and adjusted multilevel regression analysis of health centre and patient level characteristics on recording of absolute CVRA for patients with diabetes in participating health centres between 1 January 2012 and 31 December 2014 (n=121 health centres; 1,728 patient records)

Predictors		Unadjusted odds ratio			Empty Model	Model A – Health centre characteristics only			Model B – Health centre and patient characteristics		
		UOR	95%CI	p value		OR	95%CI	p value	OR	95%CI	p value
**Health Centre Characteristics**											
State	Northern Territory	127.42	48.30–336.17	0.000[b]		133.00	57.77–306.19	0.000[b]	130.33	55.45–306.35	0.000[b]
	Other states[a]	1	(base)			1	(base)		1	(base)	
Location	Non-remote	1	(base)			1	(base)		1	(base)	
	Remote	13.79	1.12–169.73	0.040[b]		1.32	9.31–5.66	0.705	1.46	0.33–6.50	0.623
	Very remote	6.83	1.17–39.93	0.033[b]		0.33	0.10–1.07	0.066	0.29	0.09–0.97	0.045[b]
Type of health centre:	Community–controlled	1	(base)			1	(base)		1	(base)	
	Government	3.02	0.72–12.55	0.129		14.61	5.62–37.95	0.000[b]	14.19	5.33–37.80	0.000[b]
Service population											
	≤500	1	(base)			1	(base)		1	(base)	
	501 – 999	0.32	0.08–1.31	0.115		0.91	0.38–2.14	0.823	0.97	0.40–2.33	0.944
	≥1000	0.27	0.08–0.89	0.032[b]		0.64	0.30–1.38	0.256	0.64	0.29–1.37	0.248
CQI experience	Nil previous cycles	1	(base)			1	(base)		1	(base)	
	1–2 previous cycles	2.19	0.33–14.42	0.416		0.66	0.21–2.09	0.479	0.58	0.18–1.89	0.368
	≥3 previous cycles	2.01	0.30–13.37	0.469		0.78	0.25–2.42	0.668	0.69	0.22–2.19	0.532
**Patient Characteristics**											
Sex	Male	1	(base)						1	(base)	
	Female	1.48	1.09–2.00	0.012[b]					1.52	1.11–2.08	0.009[b]
Age Group	15 – <30	1	(base)						1	(base)	
	30 – <45	1.07	0.64–1.80	0.791					1.07	0.64–1.80	0.797
	45 – 60	1.20	0.72–2.02	0.483					1.27	0.74–2.17	0.386
Comorbidities[c]	Hypertension	1.03	0.75–1.42	0.835					0.97	0.69–1.37	0.866
	COPD	0.94	0.60–1.47	0.793					0.88	0.56–1.39	0.592
	Dyslipidaemia	1.23	0.91–1.67	0.183					1.21	0.88–1.67	0.246
	CKD	1.55	1.08–2.22	0.017[b]					1.32	0.92–1.90	0.136
	Depression	0.81	0.42–1.55	0.527					0.89	0.45–1.76	0.737
	Other mental illness	0.70	0.34–1.45	0.337					0.74	0.35–1.55	0.420
Complications[c]	Retinopathy	0.90	0.44–1.83	0.764					0.89	0.43–1.86	0.764
	Neuropathy	0.74	0.35–1.56	0.429					0.73	0.34–1.56	0.410
	Foot ulcer	2.14	0.65–6.99	0.210					2.41	0.71–8.18	0.158
	Amputation	1.45	0.27–7.79	0.667					1.31	0.26–6.67	0.748
	Gastroparesis	1	(empty)[d]						1	(empty)[d]	
**Random effects (intercepts)**											
Variance (SE)					10.04 (2.46)	1.22	(0.35)		1.26	(0.36)	
Proportional change in variance						87.82%			87.40%		

[a]Other states include Queensland, South Australia and Western Australia

[b] Statistically significant

[c]Comorbidities and complications were compared with patients without the specific comorbidity or complication, such that an odds ratio of 1 relates to not having the specific comorbidity or complication.

[cd]The 19 patients with gastroparesis did not have any recorded CVRA.
