# Supplementary material for: Wide Variation in Absolute Cardiovascular Risk Assessment in Aboriginal and Torres Strait Islander People with Type 2 Diabetes
Source: Front Public Health. 2016 Mar 8;4:37. doi: 10.3389/fpubh.2016.00037 (PMC4781864; doi:10.3389/fpubh.2016.00037)
Supplement: Supplementary file 2 [file Data_Sheet_2.PDF]

Appendix 2: Unadjusted and adjusted multilevel regression analysis of health centre and patient level characteristics on recording of absolute CVRA for patients with diabetes and no documented chronic kidney disease in participating health centres in the Northern Territory between 1 January 2012 and 31 December 2014 (n= 52 health centres; 677 patient records)

**Predictors**		**Unadjusted odds ratio**			**Empty Model**	**Model A – Health centre characteristics only**			**Model B – Health centre and patient characteristics**		
		**UOR**	**95%CI**	**p value**		**OR**	**95%CI**	**p value**	**OR**	**95%CI**	**p value**
**Health Centre Characteristics**											
Location	Non-remote	1	(base)			1	(base)		1	(base)	
	Remote	2.69	0.17–41.48	0.478		8.18	0.92–72.79	0.060	8.51	0.86–84.67	0.068
	Very remote	2.11	0.19–22.92	0.540		1.33	0.21–8.57	0.763	1.17	0.17–8.25	0.876
Type of health centre: Community–		1	(base)			1	(base)				
controlled		11.41	4.83–26.91	0.000[a]		14.39	5.41–38.26	0.000[a]	16.05	5.72–45.01	0.000[a]
	Government										
Service population											
	≤500	1	(base)			1	(base)		1	(base)	
	501 – 999	0.45	0.14–1.44	0.177		1.44	0.54–3.88	0.470	1.47	0.52–4.11	0.464
	≥1000	0.36	0.13–1.00	0.049[a]		0.60	0.26–1.41	0.243	0.64	0.26–1.55	0.321
CQI experience	Nil previous cycles	1	(base)			1	(base)		1	(base)	
	1–2 previous cycles	3.07	0.46–20.53	0.246		1.17	0.28–4.91	0.827	1.13	0.26–5.01	0.871
	≥3 previous cycles	1.06	0.16–6.91	0.953		0.99	0.25–3.95	0.988	0.98	0.23–4.15	0.980
**Patient Characteristics**											
Sex	Male	1	(base)						1	(base)	
	Female	1.50	1.01–2.24	0.047[a]					1.48	0.97–2.25	0.068
Age Group	15 – <30	1	(base)						1	(base)	
	30 – <45	1.04	0.58–1.87	0.901					1.02	0.56–1.88	0.939
	45 – 60	1.16	0.64–2.09	0.634					1.19	0.63–2.25	0.602
Comorbidities[b]	Hypertension	1.00	0.63–1.58	0.998					0.92	0.56–1.49	0.725
	COPD	0.70	0.40–1.25	0.231					0.71	0.39–1.28	0.249
	Dyslipidaemia	1.08	0.73–1.60	0.700					1.20	0.78–1.83	0.403
	Depression	1.08	0.47–2.49	0.861					1.43	0.57–3.61	0.446
	Other mental illness	0.47	0.18–1.22	0.121					0.41	0.15–1.13	0.084
Complications[b]	Retinopathy	1.23	0.45–3.38	0.683					1.36	0.47–3.97	0.575
	Neuropathy	0.41	0.14–1.17	0.095					0.41	0.14–1.20	0.103
	Foot ulcer	2.25	0.51–10.03	0.286					2.83	0.59–13.61	0.193
	Amputation	1.31	0.18–9.43	0.789					1.07	0.14–8.40	0.947
	Gastroparesis	1	(empty)						1	(empty)	
**Random effects (intercepts)**											
Variance (SE)					2.27 (0.65)	0.81	(0.30)				
Proportional change in variance						64.27%					

[a] Statistically significant
[b] Comorbidities and complications were compared with patients without the specific comorbidity or complication, such that an odds ratio of 1 relates to not having the specific comorbidity or complication.
[c] The 5 patients with gastroparesis did not have any recorded CVRA.
